# High-Quality Draft Genome Sequence of *Francisella tularensis* subsp. *holarctica* Strain OR96-0246

**DOI:** 10.1128/genomeA.00898-15

**Published:** 2015-08-13

**Authors:** L. M. Atkins, M. E. Holder, N. J. Ajami, G. A. Metcalf, G. M. Weissenberger, M. Wang, V. Vee, Y. Han, D. M. Muzny, R. A. Gibbs, J. F. Petrosino

**Affiliations:** a Department of Molecular Virology and Microbiology, Baylor College of Medicine, Houston, Texas, USA; b Alkek Center for Metagenomics and Microbiome Research, Baylor College of Medicine, Houston, Texas, USA; c Human Genome Sequencing Center, Baylor College of Medicine, Houston, Texas, USA

## Abstract

The bacterial pathogen *Francisella tularensis* was recently renewed as a tier-one select agent. *F. tularensis* subsp. *tularensis* (type A) and *holarctica* (type B) are of clinical relevance. Here, we report the complete genome of a virulent *F. tularensis* type B strain and describe its usefulness in comparative genomics.

## GENOME ANNOUNCEMENT

*Francisella tularensis* is a Gram-negative intracellular coccobacillus and etiological agent of the zoonotic disease tularemia (1). Subspecies *tularensis* (type A) and *holarctica* (type B) are responsible for all tularemia-associated fatalities in the United States, with type A infections resulting in significantly higher mortality than type B infections (2). The Federal Select Agent Program classifies *F. tularensis* as a tier-one select agent due to its low infectious dose, high morbidity, and lack of a licensed vaccine (3, 4). An attenuated live vaccine strain (LVS) was empirically derived from repeated passage of a type B strain in the 1950s. While LVS offers less protection against inhalational type A, it is to date the only vaccine for which formal efficacy data in humans exists (5). LVS remains unlicensed, and its exact mechanism of attenuation is unknown.

Live attenuated type A strains are highly scrutinized as tularemia vaccines due to reversion concerns. Recent attempts to derive a live vaccine strain from type A were unsuccessful, as disruption of multiple loci abolished the protective ability of the attenuated strain (6–8). Therefore, virulent and potentially immunogenic type B strains are ideal sources of a live attenuated vaccine. To this end, a study reported success with an attenuated type B strain (FSC200Δ*clpB*), which afforded greater protection against respiratory SchuS4 (type A) challenge than LVS in mice (8). However, as a chaperone protein, ClpB is likely to interact with many proteins and therefore does not address specific mechanisms of *Francisella* attenuation or virulence.

Studies in type B strains offer the major advantage that new information will build off of current knowledge and positive attributes of LVS. *F. tularensis* subsp. *holarctica* strain OR96-0246 was isolated in 1996 from a primate facility outbreak in Oregon (9, 10). OR96-0246 NR-648 was obtained through BEI Resources, NIAID, NIH, and grown in cation-adjusted Mueller-Hinton (MHII) broth (Becton Dickinson) supplemented with sterile 0.1% glucose (Fisher), 0.025% ferric pyrophosphate (Sigma), and 2% reconstituted IsoVitaleX (Becton Dickinson). A total of 10 to 20 µg of high-molecular-weight genomic DNA was extracted from overnight cultures and precipitated with ethanol (11). Extracts were plated on MHII agar (Becton, Dickinson) to confirm sterility. DNA was sequenced on the Pacific Biosciences RS II platform according to the manufacturer’s protocols. Reads were fed into the Celera assembler and annotated using the NCBI tools Glimmer and GeneMark (12, 13).

We report a high-quality draft of the OR96-0246 genome as one circle, with a gap, comprising 1,879,883 bases with 32.2% GC content and an average reference coverage of 142. The postfilter read *N*_50_ is 16,096 bases, with a quality value of 0.833. Annotation features include 1,976 genes, 213 pseudogenes, 10 rRNAs, 38 tRNAs, and 1 noncoding RNA (ncRNA). Two 23-kb repetitive regions encode the *Francisella* pathogenicity island (FPI), of which one region is fully represented in the assembly. The assembly process stacked all of the internal reads of the repeat into the assembled copy, confirmed by double coverage depth (>350).

### Nucleotide sequence accession number.

The sequence of OR96-0246 has been deposited in NCBI GenBank under the accession no. CP011488.
